# Spatial Patterns of Primate Electrocutions in Diani, Kenya

**DOI:** 10.1007/s10764-018-0046-6

**Published:** 2018-07-12

**Authors:** Lydia Katsis, Pamela M. K. Cunneyworth, Katy M. E. Turner, Andrea Presotto

**Affiliations:** 1 Faculty of Health Sciences, University of Bristol, Bristol, BS8 1TH UK; 2 Colobus Conservation, Diani, 80401 Kenya; 3 Department of Geography and Geosciences, Salisbury University, Salisbury, MD 21801 USA

**Keywords:** Electrocution, GIS, Hotspots, Power lines, Spatial analysis

## Abstract

Electrocution from power infrastructure threatens many primate species, yet knowledge of effective evidence-based mitigation strategies is limited. Mitigation planning requires an understanding of the spatial distribution of electrocutions to prioritize high-risk areas. In Diani, a coastal Kenyan town, electrocution is an important cause of death for five primate species. In this study we aim to describe the spatial patterns of electrocutions and electric shock incidents (collectively referred to as electrocutions hereafter) and identify electrocution hotspots to guide an effective primate conservation approach in Diani. Colobus Conservation, a not-for-profit organization, has recorded electrocutions and annual primate census data since 1998. We georeferenced 329 electrocution data points and analyzed them using QGIS. We identified and compared hotspots across species, seasons, and time using kernel density estimation and Getis-Ord-Gi*. We employed spatial regression models to test whether primate population density and power line density predicted the location of electrocution hotspots. Electrocutions occurred in hotspots that showed little variation in location between species and seasons. The limited variation in hotspot location over time likely occurred as a result of new building development in Diani and variability in primate detection rates by community members. Primate density and power line density were significant predictors of electrocution density for Angolan black-and-white colobus (*Colobus angolensis palliatus*) and Sykes monkeys (*Cercopithecus mitis albogularis*), but the relationship was weak, suggesting the presence of additional risk factors. This study provides a framework for systematic spatial prioritization of power lines that can be used to reduce primate electrocutions in Diani, and can be adopted in other areas of the world where primates are at risk from electrocution.

**Electronic supplementary material:**

The online version of this article (10.1007/s10764-018-0046-6) contains supplementary material, which is available to authorized users.

## Introduction

Primates are at high risk of extinction as a result of unsustainable human activity, which is causing extensive habitat loss and degradation (Estrada *et al.* 2017; Rudran 2015). Consequently many species are restricted to human dominated landscapes (Arroyo-Rodríguez and Fahrig 2014), where their survival is threatened by novel risks including electrocution from overhead power lines (Parker *et al.* 2008; Ram *et al.* 2015; Slade 2016). Primates are exposed to power lines as they use them to travel across the landscape, especially between isolated tree patches, and to escape from aggressors (Boinski *et al.* 1998; Dittus 1986; Goulart *et al.* 2010; Ram *et al.* 2015). As cables are rarely insulated, this behavior poses a high risk of electrocution (lethal) or electric shock injury (not immediately lethal) (Dwyer 2006) when individuals simultaneously grasp two conductors, or a conductor and an earthed device (Bevanger 1998). Similarly, injury can result from wet vegetation contacting energized components, creating short circuits from power lines to the ground (Kumar and Kumar 2015). Reported death rates after electric shock range from 31% to 36% (Kumar and Kumar 2015; Slade 2016), with individuals dying from the effects of electric current passing through the body (Schulze *et al.* 2016), or from the subsequent impact of falling from a height (Kumar and Kumar 2015). Survivors of electric shocks are frequently left with injuries to the hands, head, and chest, and may later die from secondary infection (Kumar and Kumar 2015). In addition, these incidents cause power outages, equipment damage, and fires, which affect human communities (APLIC 2006; Dwyer *et al.* 2014; Harness and Wilson 2001; Printes 1999).

Electrocutions are documented for a range of primate species across Asia (Kumar and Kumar 2015; Nekaris and Jayewardene 2004; Roscoe *et al.* 2013), Africa (Maibeche *et al.* 2015; Slade 2016), and Latin America (Goulart *et al.* 2010; Printes 1999; Rodrigues and Martinez 2014). They are a principal mortality factor for the Endangered Central American squirrel monkey subspecies *Saimiri oerstedii citrinellus* and *Saimiri oerstedii oerstedii* (Boinski *et al.* 1998), and were found to be the most common cause of death for a population of Hanuman langurs (*Semnopithecus entellus*) (Ram *et al.* 2015). There is limited knowledge about population-level effects of electrocution on primates, but avian studies have shown that even low electrocution rates can drive declining populations to local extinction (Hernández-Matías *et al.* 2015). Therefore, evidence of electrocutions of Critically Endangered species including the Javan slow loris (*Nycticebus javanicus*) (Moore and Nekaris 2014) and the western purple-faced langur (*Trachypithecus vetulus nestor*) (Moore *et al.* 2010; Parker *et al.* 2008) is a cause for conservation concern.

As habitat encroachment increases and power line networks rapidly expand, this problem is likely to escalate in the future (Bevanger 1994; Jenkins *et al.* 2010), and development of effective evidence-based mitigation strategies is crucial (Sutherland *et al.* 2004). Current strategies to reduce electrocution include power line insulation, tree-trimming around power lines, artificial canopy bridges, and braiding of power lines (Lokschin *et al.* 2007; Printes 1999; Roscoe *et al.* 2013); however, their effectiveness in reducing electrocutions is rarely evaluated (Teixeira *et al.* 2013). Limited funding makes mitigation measures across the entire power grid unfeasible; therefore measures must be targeted to high-risk areas (Dwyer *et al.* 2014; Lokschin *et al.* 2007). This requires an understanding of the spatial distribution of electrocutions (Guil *et al.* 2011; Malo *et al.* 2004), which is rarely studied for primates.

In the Kenyan town of Diani, electrocution contributes to mortality for five of the six primate species: Angolan black-and-white colobus (*Colobus angolensis palliatus*), Sykes monkeys (*Cercopithecus mitis albogularis*), vervet monkeys (*Chlorocebus pygerythrus hilgerti*), northern yellow baboons (*Papio cynocephalus ibeanus*), and white-tailed small-eared galagos (*Otolemur garnettii lasiotis*). No electrocutions have been recorded for the Kenya coast dwarf galago (*Paragalago cocos*), possibly because of their small body size (Slade 2016). Angolan black-and-white colobus monkeys are particularly affected, with annual mortality estimates ranging from 1.7% to 7.9% (Slade 2016). We aim to describe the spatial patterns of primate electrocutions and electric shock incidents (collectively referred to as electrocutions hereafter) in Diani and to identify electrocution hotspots to inform an effective evidence-based mitigation strategy. Our hypothesis is that particular locations will be more likely to result in reported electrocutions than others, owing to landscape features (e.g., proximity of power lines to trees), behavioral factors (e.g., habitually used routes), or demographic factors (e.g., locations with higher densities of primates). We predict that 1) electrocutions will occur in hotspots, 2) hotspots will be species specific, 3) hotspots will differ between seasons, 4) hotspots will change over time, 5) hotspots will be associated with high primate density, and 6) hotspots will be associated with high power line density.

## Methods

### Study Site

**Fig. 1:**
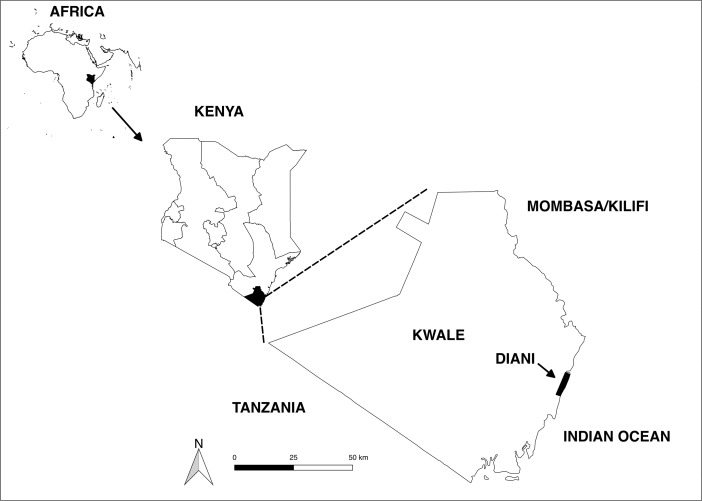
Map of southern Kenya, indicating location of study site (Diani).

Diani is a touristic coastal town in southern Kenya, located 30 km south of Mombasa in Kwale County (Fig. 1). It is in the fragmented Diani Forest, a narrow strip of coastal rag forest *ca*. 10 km long by 0.5 km wide (Dunham 2014). This forest is part of the Zanzibar–Inhambane Undifferentiated Floristic Region (White 1983), a global biodiversity hotspot undergoing extensive habitat loss (Brooks *et al.* 2002; Myers *et al.* 2000). Rainfall is bimodal, with long rains occurring from April to June and short rains from October to November (Colobus Conservation, *unpubl. data*).

As a result of expansive human development in Diani, a high proportion of forest cover has been lost and the remaining forest is highly fragmented by roads, resorts, developed land, and overhead power lines (Kanga and Heidi 1999; Moreno-Black and Maples 1977). Consequently, primates are often threatened by human activity, and a local conservation organization, Colobus Conservation, operates a primate rescue center in this area.

### Data Collection

Kenya Power provided the power infrastructure data in digital CAD format (an image file format used by the software AutoCad). We extracted from the CAD files all above ground power lines within the study area and verified power line location against logical pathways down major highways and against shadows cast in high-resolution imagery in nonpopulated places. We also tracked the power infrastructure the power lines entered and exited, such as substations, from the imagery and the CAD file. We converted the power line data from the provided projection EPSG:21307 to the more standard EPSG:32737, which is compatible with current GPS standards. We used the Kenya datum conversion information provided by the American Society for Photogrammetry and Remote Sensing (ASPRS 1983) to complete this transformation.

Colobus Conservation provided electrocution and electric shock data from 1998 to the end of 2016. Community members report welfare cases and dead animals to the Colobus Conservation emergency response team, which attends to the cases and records data. Data include each reported electrocution case, primate species, description of the incident, and incident GPS location since 2010. Colobus Conservation logs these data onto a record sheet and enters it into an electronic database. We collated and crosschecked welfare reports against the electronic database to compile a dataset of 370 electrocution incidents. We validated all location coordinates against a base map and updated incorrect locations. Of 370 incident reports, 266 were associated with a specific location and valid GPS location (Garmin eTrex 30×); 63 only provided the name of the property where the incident occurred, so we took coordinates for all power lines associated with the property, and assigned a random point to the power line using the QGIS random points tool; the remaining 41 reports did not contain sufficient information to assign a GPS location and we excluded them from the analysis.

Since 1997 Colobus Conservation has conducted annual primate censuses in Diani for northern yellow baboons, Sykes monkeys, vervet monkeys, and Angolan black and white colobus, and provided this population data for our study. They did not collect census data for either galago species. They conduct the census each year in October. For northern yellow baboons, Colobus Conservation visited each known group in Diani three to six times within a week, and determined the group size as the mode of repeated counts. They calculated the census figure as the total of all groups. For Sykes monkeys, vervet monkeys, and Angolan black-and-white colobus, they conducted line transect surveys throughout every property in Diani and the remaining forest over 3–4 days. At each plot, observers spread themselves at intervals and moved along a prescribed route at 1–1.5 km per hour, stopping periodically to watch and listen for primates. Distance between observers varied at each plot depending on the density of the foliage, but ranged from 10 to 200 m. They walked transects in east to west direction, and then returned in west to east direction until they covered the entire plot. When they encountered a group of monkeys, observers joined together and recorded the GPS location, time of discovery, species, number of primates, sex composition, age composition, and direction of movement. They then returned to their last survey mark to complete the census walk. The conservation manager checked each data sheet to identify any repeated counts. The observers determined the census figure as the total of these raw counts. As the primates in Diani are habituated, the observers recorded data at distances of 10–20 m from the primates. We checked the coordinates of census group locations against a base map to identify incorrect locations, and removed data from years with inaccurate readings**.** This left 10 years of accurate census data for Angolan black-and-white colobus, 9 years of accurate data for vervet monkeys, and 8 years of accurate data for Sykes monkeys.

### Statistical Analysis

#### Electrocution Hotspots

To visualize electrocution hotspots we used two techniques: 1) kernel density estimation (KDE) and 2) Getis-Ord-Gi*. KDE is a common technique used in ecological studies (Kernohan *et al.* 2001), and is used to identify wildlife road traffic accident hotspots (Gomes *et al.* 2009). We implemented KDE using the QGIS Heatmap plugin, which creates a density surface of the electrocution points based on the number of points per unit area. A moving function weights points within a region of influence based on the distance of each point to the location of interest. The area of influence is determined by the bandwidth, with larger bandwidths resulting in a smoother surface (Gatrell *et al.* 1996). We used a bandwidth of 500 m for each heatmap, as it enabled good resolution of hotspots and allowed for comparison between data subsets. We then visualized hotspots for all electrocution records, for individual species, and for different seasons. The KDE algorithm we used was developed for planar analysis, but the dataset we analyzed occurred along the power line network. Therefore we performed an additional KDE developed for network analysis using the v.kernel tool from GRASS GIS (GRASS Development Team 2017). For this analysis we used the split nodes method and the suggested bandwidth of 10 map units, and multiplied the result by the number of input points as specified in the user manual (GRASS Development Team 2018).

As KDE does not provide a measure of statistical significance of hotspots, we used the QGIS Hotspot Analysis plugin to calculate Getis-Ord-Gi* statistics (Getis and Ord 1992; Oxoli *et al.* 2016). We aggregated electrocution points into a 150 m by 150 m grid, with each cell containing a value representing the number of electrocutions. To account for the network nature of power lines and the tendency of electrocution events to cluster around them we applied Getis-Ord-Gi* using two methods. For the first method (hereafter Getis-Ord-Gi*_1_) we removed all cells that were not intersected by power lines and performed the hotspot analysis only along the power line network. This decreased the sample size, as some of the electrocution points fell outside these cells. For the second method we performed hotspot analysis along the power line network and corrected for the power line length in each cell. We calculated the linear distance of power lines in each cell (network length of power lines for each cell) and divided the number of electrocutions by the length of power lines per cell. We compared these two methods by assessing the percentage of electrocutions occurring in the resulting hotspots, and found that Getis-Ord-Gi*_1_ accounted for a higher percentage of electrocutions. Therefore for the remainder of the analysis we used only the Getis-Ord-Gi*_1_ method.

The hotspot analysis finds clustering by comparing values of each cell and its neighbors to the sum of all cells. Resultant *Z* scores give a measure of clustering, with large positive *Z* scores indicating clustering of large values (hotspot) and large negative *Z* scores indicating clustering of small values (coldspot). We overlaid hotspots identified by planar KDE and Getis-Ord-Gi* onto the power line map to calculate the percentage of power lines and electrocutions each hotspot is associated with.

#### Comparison of Electrocution Hotspots Between Species and Seasons

To compare electrocution hotspots between species and seasons, we used the planar KDE and Getis-Ord-Gi*_1_ outputs. We used the planar KDE results as opposed to those from the network KDE, as they allowed for better visualization of overlap of hotspots. Using the KDE, we divided each heatmap into five equal interval levels of density and extracted the highest two levels to create a vector outline of hotspots. We overlaid the hotspot outlines for each species onto one map to visualize hotspot overlap, and repeated this to visualize overlap between seasons.

To quantitatively assess similarity between the locations of hotspots we transformed the Getis-Ord-Gi*_1_ output into a binary variable representing electrocution hotspot presence or absence, including only hotspots with *P* ≤ 0.05 in the hotspot presence category. With these data we used Pearson’s correlation tests in R. 3.2.3 (R Core Team 2015) to test the association between species-specific hotspots and between seasonal hotspots (Teixeira *et al.* 2017).

#### Electrocution Hotspots Over Time

To assess change in electrocution rate over time, we performed an ordinary least squares regression (OLS) of yearly electrocution rate against year using R 3.2.3. To account for varying population sizes, we repeated the OLS using electrocutions year^−1^ population^−1^ for each species excluding the white-tailed small-eared galago, for which we did not have census data.

To visualize change in electrocution hotspots over time, we divided the dataset into three study periods: 1998–2003, 2004–2009, and 2010–2016. We chose these study periods to illustrate changes over time, while retaining sufficient electrocution events in each approximately equal time period. We created a KDE overlay map of hotspots as described in the foregoing for species and seasons.

#### Electrocution Hotspots and Primate and Power Line Density

To assess whether electrocution density was associated with primate density and power line density we applied spatial regression analyses using GeoDa 1.10 (Anselin *et al.* 2006). We could assess only Angolan black-and-white colobus, Sykes monkeys, and vervet monkeys, as georeferenced census data were not available for northern yellow baboons and white-tailed small-eared galagos. For each species we aggregated census and electrocution data onto a 150 m by 150 m grid, and calculated the mean density of monkeys per cell across the total number of years of census data, and the mean density of electrocutions per cell across all years. We calculated the density of power lines within each cell by dividing the length of power line by the area of each cell. We removed all cells that did not have power lines to restrict the analysis to the power line network. Because the spatial autocorrelation violates the independence assumption of OLS, we employed spatial regression models using the maximum likelihood approach and a queen’s contiguity spatial weights matrix and selected the most appropriate models based on Akaike’s information criterion (AIC), which gives a measure of relative fit of statistical models (Akaike 1974). We compared the spatial lag and spatial error models for each species because both improved the original OLS model, and consequently selected spatial lag models for Angolan black-and-white colobus and Sykes monkeys, and a spatial error model for vervet monkeys. The specification of the spatial lag model is given by

$$ \gamma =\beta o+ X\beta +\rho W\gamma +\varepsilon $$

In this model the values of the dependent variable in neighboring locations (*Wγ*) are included as an extra explanatory variable or the “spatial lag” of *γ*. The second model used, the spatial error model, is given by

$$ \gamma =\beta o+ X\beta +\rho W\varepsilon +\varepsilon $$

In this model the values of the residuals in neighboring locations (*Wε*) are included as an extra term in the equation, which are considered the “spatial error.” *W* is the spatial weights matrix in both models.

To visualize the relationship we overlaid electrocution hotspots identified by planar KDE onto a heatmap of mean population density for each species.

#### Data Availability

The datasets analyzed during the current study are deposited and available from: https://mdsoar.org/handle/11603/10940.

## Ethical Note

The Kenyan government granted permission to conduct this study (permit number NACOSTI/P/17/46068/16586). All research protocols reported in this article were reviewed and approved by the Animal Welfare Ethical Review Body at the University of Bristol, Bristol, UK.

The authors declare that they have no conflict of interest.

## Results

In Diani, between 1998 and the end of 2016, community members reported 370 electrocutions, 329 of which we georeferenced. The most commonly observed species was Angolan black-and-white colobus, followed by Sykes monkeys, white-tailed small-eared galagos, vervet monkeys, and northern yellow baboons (Table I).

**Table I:** Hotspots of primate electrocutions reported in Diani, Kenya between January 1998 and December 2016

Group	Number of electrocutions	Percentage of total electrocutions for each species	Getis-Ord-Gi*_1_(95%) hotspots		Planar kernel density estimate hotspots		
			Percentage of electrocutions occurring in hotspots	Percentage of power grid in hotspots	Number of hotspots	Percentage of electrocutions occurring in hotspots	Percentage of power grid in hotspots
All species	329	100	56	10	5	51	10
Northern yellow baboon							
*Papio cynocephalus ibeanus*	7	2	100	6	1	57	1
Angolan black-and-white colobus							
*Colobus angolensis palliatus*	232	71	58	9	4	63	13
Sykes monkey							
*Cercopithecus mitis albogularis*	47	14	73	11	8	60	6
White-tailed small-eared galago							
*Otolemur garnettii lasiotis*	31	9	53	9	5	68	9
Vervet monkey							
*Chlorocebus pygerythrus hilgerti*	12	4	50	4	2	42	1

We calculated the percentage of reported electrocutions occurring within hotspots on the power grid using two clustering methods: Getis-Ord-Gi*_1_, which does not correct for power line length, and planar kernel density estimation

We found that the 54% of cells had no power lines, hence a network distance of 0 m. Of all cells with power lines, the mean length was 200 ± SD 156 m, with a maximum power line length of 738 m. The power line lengths were longer in urbanized areas and near major roads.

### Electrocution Hotspots

**Fig. 2:**
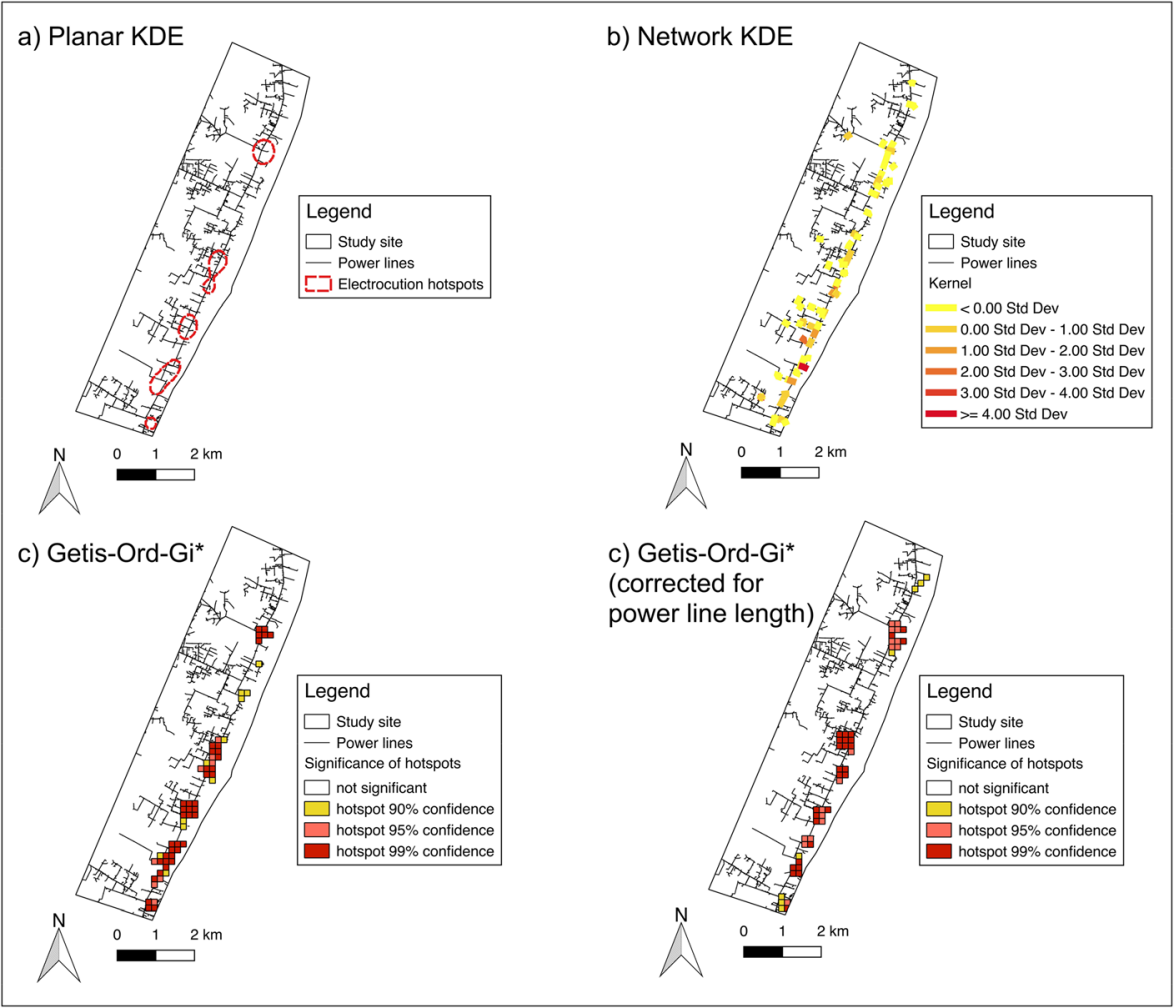
Electrocution hotspots identified for primate species in Diani, Kenya, 1998–2016, using four different methods.

Across all species, planar KDE showed that 51% of electrocutions occurred within hotspots on 10% of the power line network (Table I, Fig. 2). We could not calculate percentage of electrocutions or power line network percentages from the results of the network KDE (Fig. 2). Getis-Ord-Gi*_1_, which did not correct for power line length, showed that 56% of electrocutions occurred within hotspots on 10% of the power line network (Table I, Fig. 2). The second Getis-Ord-Gi* method, which corrected for power line length within each cell, showed that 36% of electrocutions occurred within hotspots on 7% of the power line network (Fig. 2).

**Fig. 3:**
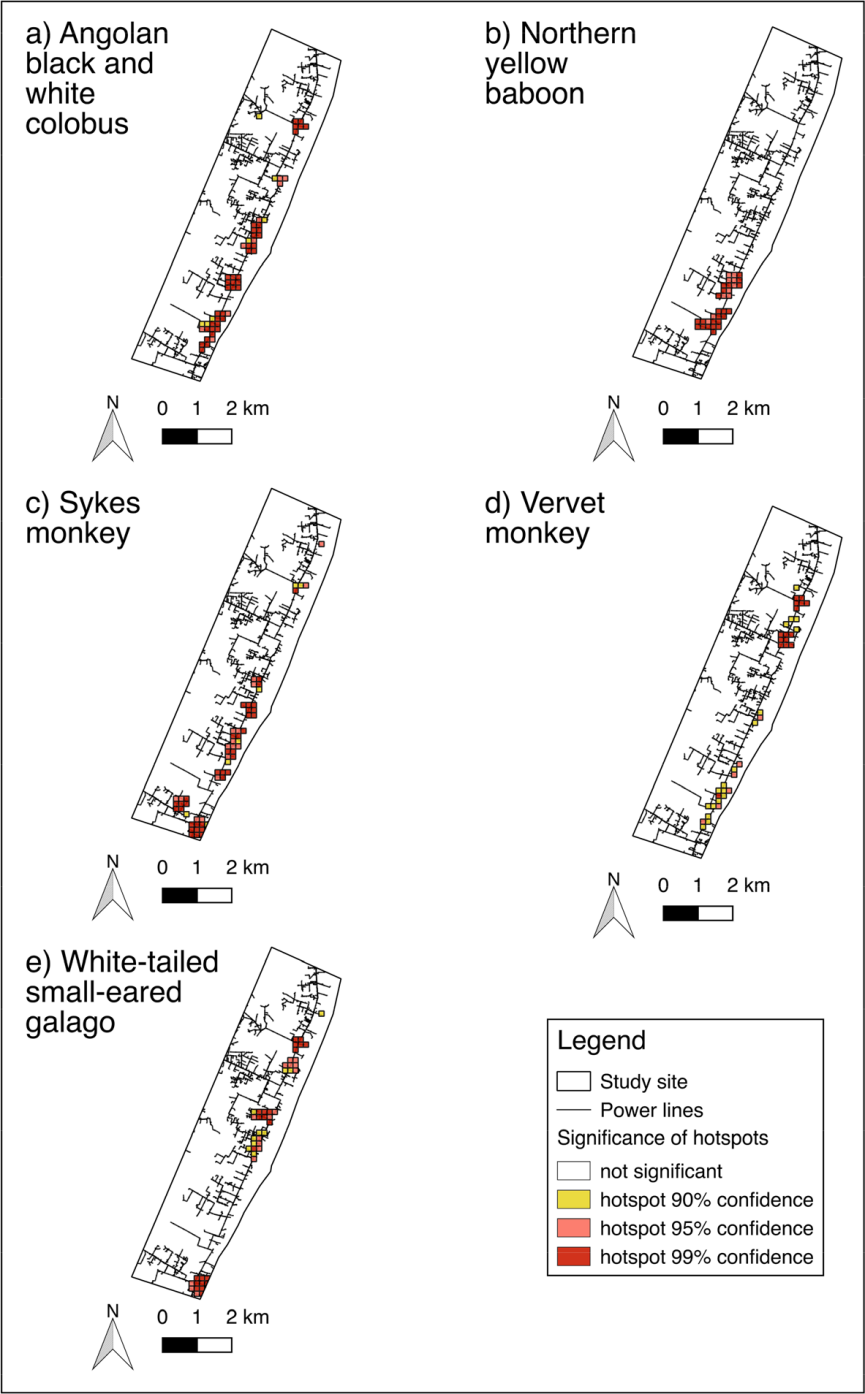
Electrocution hotspots identified by Getis-Ord-Gi*_1_ for five primate species in Diani, Kenya, 1998–2016. Getis-Ord-Gi*_1_ does not account for power line length.

Hotspots for individual species identified by Getis-Ord-Gi*_1_ (Fig. 3) largely overlapped the hotspots for all species (Fig. 2). The majority of reported electrocutions occurred along a small proportion of the power grid (Table I).

### Comparison of Electrocution Hotspots Between Species

**Fig. 4:**
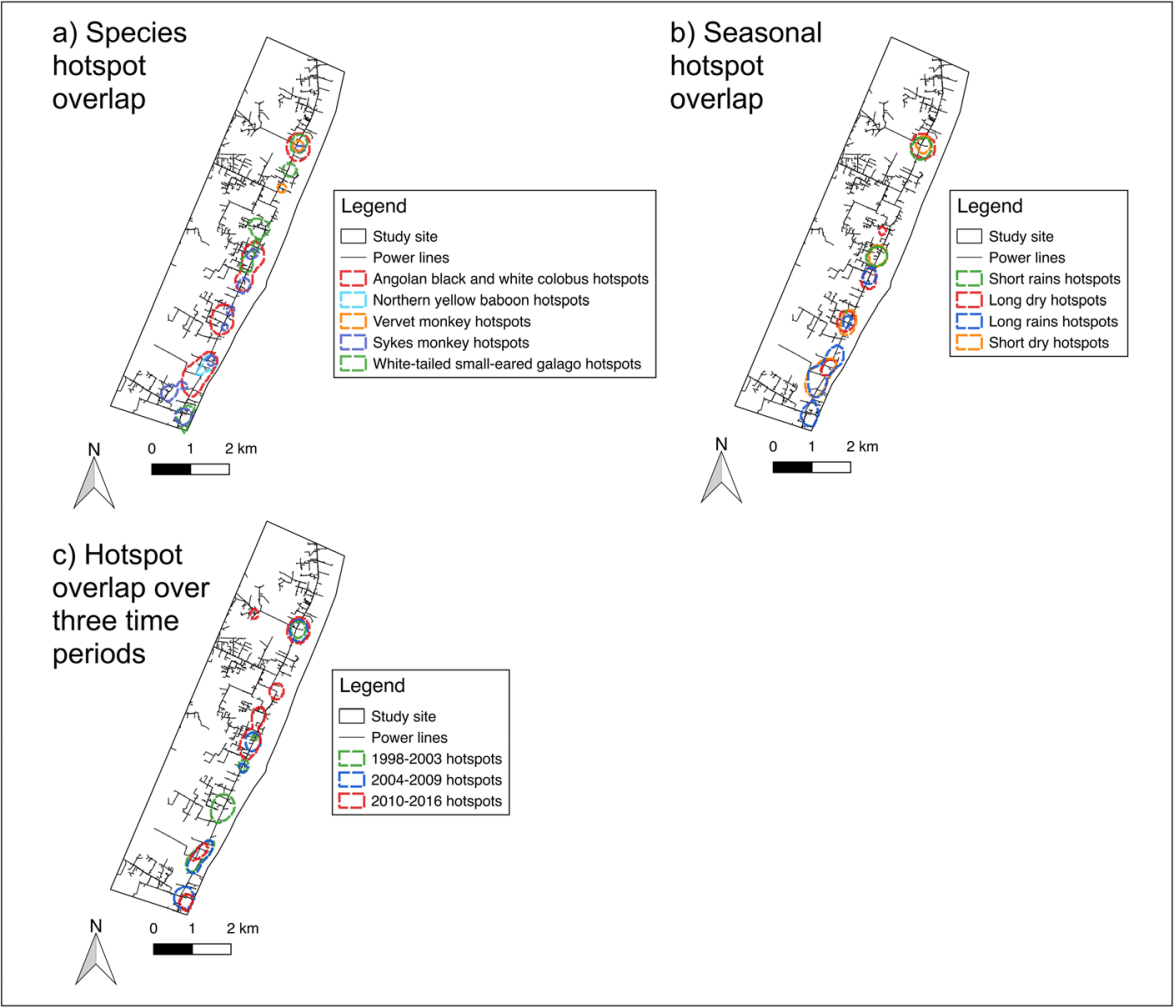
Maps showing overlay of primate electrocution hotspots identified by planar kernel density estimation for species, seasons, and over time, in Diani, Kenya between 1998 and 2016.

The hotspot overlay map shows high overlap of electrocution hotspots between different species, excluding one Sykes monkey, one vervet monkey, and two white-tailed small-eared galago hotspots that are isolated (Fig. 4). The most northerly hotspot shows high overlap between all species except northern yellow baboons. Pearson’s coefficients showed small to medium similarity for most species hotspots (Table II).

**Table II:** Results of Pearson’s correlations between hotspots of primate electrocutions reported for five primate species in Diani, Kenya, 1998–2016

	Angolan black-and-white colobus	White-tailed small-eared galago	Sykes monkey	Vervet monkey
Northern yellow baboon	*r* = 0.50 *P* < 0.001	*r* = −0.07 *P* = 0.103	*r* = 0.25 *P* < 0.001	*r* = 0.21 *P* < 0.001
Angolan black-and-white colobus	–	*r* = −0.01 *P* = 0.901	*r* = 0.27 *P* < 0.001	*r* = 0.33 *P* < 0.001
White-tailed small-eared galago	–	–	*r* = 0.26 *P* < 0.001	*r* = 0.04 *P* = 0.316
Sykes monkey	–	–	–	*r* = 0.24 *P* < 0.001

Data are for 549 grid cells across the power grid, coded as hotspot presence/absence based on Getis-Ord-Gi*_1_ (*P* ≤ 0.05) for each species

### Comparison of Seasonal Electrocution Hotspots

We observed electrocutions at a rate of 2.22 per month during the short dry season, 1.46 per month during the long rains, 1.96 per month during the long dry season, and 1.44 per month during the short rains. Seasonal hotspots showed high overlap between seasons (Fig. 4). Pearson’s coefficients showed high similarity between hotspots for the short dry season and long dry season, long rains and short dry season, and long rains and long dry season (Table III). Similarity was low between hotspots for short rains and short dry season, and there was no relationship between hotspots for the short rains and long rains (Table III). Pearson’s coefficients indicated no significant correlation between short rains and long rains hotspots (Table III).

**Table III:** Results of Pearson’s correlations between season-specific hotspots of reported primate electrocutions in Diani, Kenya, 1998–2016

	Long rains	Short dry season	Short rains
Long dry season	*r* = 0.51 *P* < 0.001	*r* = 0.73 *P* < 0.001	*r* = 0.04 *P* = 0.409
Long rains	–	*r* = 0.58 *P* < 0.001	*r* = 0.109 *P* = 0.011
Short dry season	–	–	*r* = 0.29 *P* < 0.001

Data are for 549 grid cells across the power grid, coded as hotspot presence/absence based on Getis-Ord-Gi*_1_ (*P* ≤ 0.05) for each season

### Changes over Time

Electrocutions occurred at a mean rate of 20.45 per year. OLS regression showed no statistically significant relationship in yearly rate (coefficient = 0.36, *R*^2^ = 0.078, df = 18, *P* = 0.1). OLS of electrocutions year^−1^ population^−1^ for Angolan black-and-white colobus, Sykes monkeys, vervet monkeys, and northern yellow baboons also showed no trend (Angolan black-and-white colobus: coefficient = 0.0014, *R*^2^ = 0.099, df = 9, *P* = 0.4, Sykes monkey: coefficient = 0.00020, *R*^2^ = 0.20, df = 8, *P* = 0.2, vervet monkey: coefficient = −0.00011, *R*^2^ = 0.0052, df = 8, *P* = 0.8, northern yellow baboon: coefficient = −0.00013, *R*^2^ = 0.048, df = 9, *P* = 0.5).

The hotspot overlay map (Fig. 4) showed relatively consistent overlap of hotspots since 1998. One hotspot from 1998 to 2003 has disappeared, one has increased in area, and between 2010 and 2016 two new hotspots appeared.

### Association of Electrocution Hotspots with Primate Population Density and Power Line Density

**Fig. 5:**
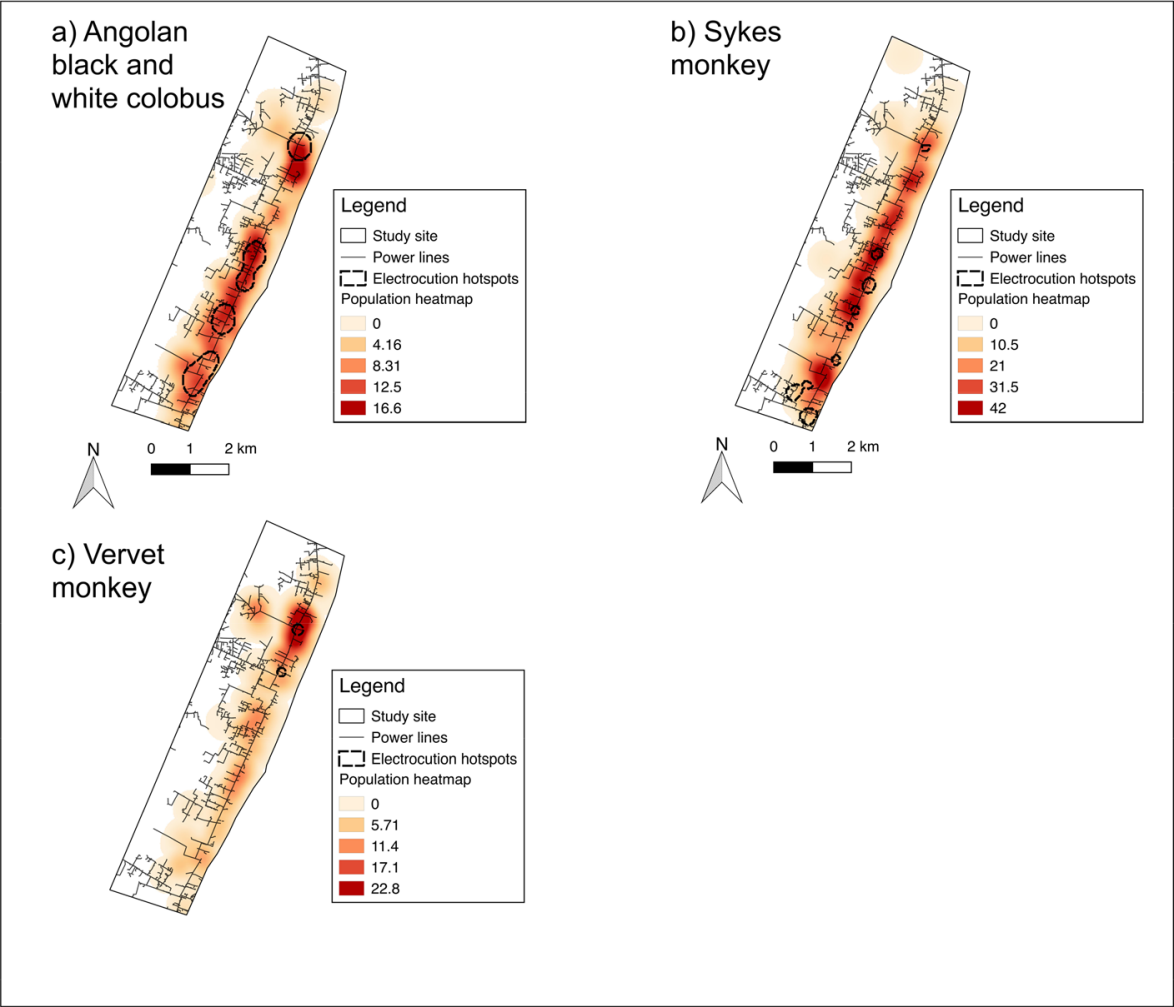
Map showing overlay of electrocution hotspots and mean density of individuals calculated by planar kernel density estimation for three species of primate in Diani, Kenya, 1998–2016

For Angolan black-and-white colobus and Sykes monkeys, spatially weighted regression models indicated a positive association between primate density and electrocution density, and power line density and electrocution density (Table IV). Overlay of electrocution hotspots onto primate density KDE showed that hotspots generally coincide with areas of high primate density (Fig. 5). However, some areas of high primate density coincide with power lines and are not associated with electrocution hotspots.

**Table IV:** Results of spatially weighted regression for primate electrocutions reported in Diani, Kenya, 1998–2016

Species	Angolan black-and-white colobus	Sykes monkey	Vervet monkey
Regression model:	Spatial lag	Spatial lag	Spatial error
Covariate:			
Mean density of individuals per cell	0.013 *P* < 0.001	0.0017 *P* < 0.001	0.00064 *P* = 0.1
Power line density	6.44e-05 *P* = 0.002	1.31e-05 *P* = 0.007	2.72e-06 *P* = 0.3
*R*^2^	0.086	0.086	0.0078
Degrees of freedom	545	545	546

Species included are Angolan black-and-white colobus (*Colobus angolensis palliatus*), Sykes monkey (*Cercopithecus mitis albogularis*), and vervet monkey (*Chlorocebus pygerythrus hilgerti*), with mean density of electrocutions per cell as the dependent variable and mean density of individuals per cell and power line density per cell as the explanatory variables

## Discussion

Primate electrocutions are not randomly distributed across the landscape in Diani. Electrocutions occur in hotspots that show little variation in location between species and seasons. Yearly electrocution rate is stable but the location of hotspots has changed over time. Primate density and power line density are associated with hotspots, but the low *R*^2^ values suggest the presence of additional risk factors, such as power line structure and environmental factors.

Hotspots identified in this study show that most primate electrocutions occurred on a small proportion of the power grid. This pattern is similar to that reported for Hanuman langurs in India, where a high incidence of electrocutions occurred in one location at the same power pole (Ram *et al.* 2015), and is commonly observed in avian studies (Dwyer *et al.* 2014; Guil *et al.* 2011; Mañosa 2001; Tintó *et al.* 2010).

We found that planar KDE and Getis-Ord-Gi*_1_ were the most practical methods to test for the presence of hotspots in our study. The output from network KDE identified specific sections of power line with high electrocution density, whereas the planar KDE identified broader, discrete areas of high electrocution density in Diani. Although applying planar KDE to a network-based dataset may produce biased estimates (Okabe *et al.* 2009), we found that hotspots from the planar KDE showed high overlap with hotspots from the Getis-Ord-Gi*, which did account for the network nature of the dataset. For Getis-Ord-Gi*, we found that accounting for power line length within each cell resulted in areas of high electrocution density with high power line density being omitted from the hotspot map, suggesting that electrocution risk is greater in areas with high power line density. Identifying only hotspots in areas with low power line density is not practical for our purpose, as areas where high electrocution density coincides with high power line density should also be prioritized for mitigation.

Understanding how species hotspots relate to each other is important to ascertain whether species-specific mitigation strategies are required (Teixeira *et al.* 2017). The Pearson’s correlation suggested small to medium similarity between most species-specific hotspots, but overlay of planar KDE hotspots showed substantial overlap for most species. The Pearson’s correlation may have identified lower similarity between hotspots, as this method examines the association of hotspots between each pair of species, whereas the planar KDE examines the overlap between all the species. Consequently the planar KDE demonstrated a higher degree of overlap, as a high proportion of species-specific hotspots fall within the Angolan black-and-white colobus hotspots, as this species has the most extensive electrocution hotspots. Similarity between species hotspots is unsurprising as the primate species in Diani have overlapping ranges (Moreno-Black and Maples 1977), and many of them have been documented to interact with each other (De Jong and Butynski 2010; Moreno-Black and Maples 1977). Frequent association and overlapping ranges are likely to lead to similarities in location of electrocutions between the species.

The species-specific hotspots generally fall within the planar KDE hotspots created for all species. This suggests a general, non–species-specific approach would be beneficial for most primates in Diani, but a species-specific approach may be needed to further reduce Sykes monkey, vervet monkey, and white-tailed small-eared galago electrocutions.

Electrocution rate increased during the dry seasons, but the locations of electrocution hotspots showed minimal variation between the seasons. This suggests variations in seasonal risk do not usually need to be factored into prioritization of areas for mitigation interventions in Diani. In contrast to our results, higher risk of electrocution is typically associated with wet seasons, as shown for raptors (Olendorff *et al.* 1981), Asian elephants (*Elephas maximus*: Palei *et al.* 2014), and rhesus macaques (*Macaca mulatta*: Kumar and Kumar 2015). A proposed explanation for elevated risk during the dry season in Diani is increased vegetation growth toward the start of the long dry season, resulting in increased contact between trees and power lines (Slade 2016). An alternative explanation is seasonal use of power lines by monkeys, possibly linked to the availability of food resources (Lokschin *et al.* 2007).

Since 1998 yearly electrocution rates have remained stable, but the location of hotspots has varied. Changes in location of hotspots may be due to the rapid development of Diani, which has resulted in the construction of new power infrastructure and ultimately in changes to food resource distribution. Alternatively, these changes may be associated with variable detection rates in some areas owing to the movement of people or urbanization. For instance, a hotspot from 1998 to 2003 that has disappeared is associated with an abandoned hotel that burnt down in 1998 (Colobus Conservation, *pers. comm*. 2017); therefore electrocutions are unlikely to be reported. Owing to these confounding factors it is important to include all available data from the whole study period to make an informed decision on mitigation strategies (Eberhardt *et al.* 2013).

Electrocution hotspots are associated with primate density and power line density for Sykes monkeys and Angolan black-and-white colobus. Additional risk factors that were not included in our study may be associated with power line structure and environmental factors such as vegetation cover and food abundance, as shown in avian studies (Dwyer *et al.* 2014; Guil *et al.* 2011; Mañosa 2001; Tintó *et al.* 2010). It is important to identify these risk factors to identify high-risk areas before electrocutions occur, and target these areas for mitigation (Dwyer *et al.* 2014; Shaw *et al.* 2010). In addition, we showed that there are specific areas where high primate density coincides with power line infrastructure, yet not associated with electrocution hotspots, suggesting that there may be protective factors at these sites. It may be helpful to assess potential protective factors in these locations, which could include a behavioral change of primates (avoidance), or fewer environmental and structural risk factors.

Limitations of this study primarily relate to data collection techniques. As Colobus Conservation relies on reports from community members, incidents in inaccessible areas are likely to go underreported, while incidents close to the main road are more likely to be reported. Furthermore, the movement of wounded animals may affect data collection (Bevanger 1999). The dataset is composed of all known electrocution and electric shock cases, including incidents that were directly observed, where the primate was found attached to the power line, and those found away from power lines, dead or alive. We do not know how far primates who have electric shock injuries travel, but we assume that the location of individuals that survive the initial injury provides a good indication of the area that they were electrocuted. Despite these limitations, this study benefits from a dataset spanning 18 years, which increases the reliability of using hotspots to guide mitigation strategies (Eberhardt *et al.* 2013).

Electrocution is an issue for many threatened primate species, yet the development of effective evidence-based mitigation strategies is limited. This study provides a framework for systematic spatial prioritization of high-risk areas that will contribute to more effective mitigation planning. This framework can be used across the world to understand and reduce primate electrocutions. Future studies should aim to objectively evaluate and compare current mitigation measures, especially comparing fatalities before and after. Furthermore, electrocution hotspots should be profiled to identify risk factors such as habitat and high-risk power line components, to guide a proactive mitigation approach that aims to reduce the risk before mortality has occurred.

## Electronic supplementary material

ESM 1 (DS_STORE 6 kb)
